# Association of Short-Term Particulate Matter Exposure among 5-Year Cancer Survivors with Incident Cardiovascular Disease: A Time-Stratified Case-Crossover Study

**DOI:** 10.3390/ijerph18157996

**Published:** 2021-07-28

**Authors:** Seulggie Choi, Kyae Hyung Kim, Daein Choi, Seogsong Jeong, Kyuwoong Kim, Jooyoung Chang, Sung Min Kim, Seong Rae Kim, Yoosun Cho, Gyeongsil Lee, Joung Sik Son, Sang Min Park

**Affiliations:** 1 Department of Biomedical Sciences, Graduate School, Seoul National University, Seoul 03080, Korea; seulggie@gmail.com (S.C.); seogsongjeong@gmail.com (S.J.); joomyjoo@gmail.com (J.C.); ksm9904@naver.com (S.M.K.); 2 Department of Family Medicine, Seoul National University Hospital, Seoul 03080, Korea; truwhat@gmail.com (K.H.K.); gespino1.gs@gmail.com (G.L.); 3 Department of Internal Medicine, Mount Sinai Beth Israel, Icahn School of Medicine at Mount Sinai, New York, NY 10029, USA; daeinchoi.md@gmail.com; 4 National Cancer Control Institute, National Cancer Center, Goyang 10408, Korea; kwkim238@gmail.com; 5 College of Medicine, Seoul National University Hospital, Seoul 03080, Korea; sungkim20@snu.ac.kr; 6 Total Healthcare Center, Kangbuk Samsung Hospital, School of Medicine, Sungkyunkwan University, Seoul 06351, Korea; misslonghorn46@gmail.com; 7 Department of Family Medicine, Korea University Guro Hospital, Seoul 08308, Korea; medical114@naver.com; 8 Department of Biomedical Sciences, College of Medicine, Seoul National University, Seoul 03080, Korea

**Keywords:** particulate matter, cancer survivors, cardiovascular disease

## Abstract

The association of short-term particulate matter concentration with cardiovascular disease (CVD) among cancer survivors is yet unclear. Using the National Health Insurance Service database from South Korea, the study population consisted of 22,864 5-year cancer survivors with CVD events during the period 2015–2018. Using a time-stratified case-crossover design, each case date (date of incident CVD) was matched with three or four referent dates, resulting in a total of 101,576 case and referent dates. The daily average particulate matter 10 (PM10), 2.5 (PM2.5), and 2.5–10 (PM2.5–10) on the day of case or referent date (lag0), 1–3 days before the case or referent date (lag1, lag2, and lag3), and the mean value 0–3 days before the case or referent date (lag0–3) were determined. Conditional logistic regression was conducted to calculate the adjusted odds ratios (aORs) and 95% confidence intervals (CIs) for CVD according to quartiles of PM10, PM2.5, and PM2.5–10. Compared to the 1st (lowest) quartile of lag0–3 PM10, the 4th (highest) quartile of lag0–3 PM10 was associated with higher odds for CVD (aOR 1.13, 95% CI 1.06–1.21). The 4th quartiles of lag1 (aOR 1.12, 95% CI 1.06–1.19), lag2 (aOR 1.09, 95% CI 1.03–1.16), lag3 (aOR 1.06, 95% CI 1.00–1.12), and lag0–3 (aOR 1.11, 95% CI 1.05–1.18) PM2.5 were associated with higher odds for CVD compared to the respective 1st quartiles. Similarly, the 4th quartile of lag0–3 PM2.5–10 was associated with higher CVD events (aOR 1.11, 95% CI 1.03–1.19) compared to the 1st quartile. Short-term exposure to high levels of PM may be associated with increased CVD risk among cancer survivors.

## 1. Introduction

Particulate matter (PM), minute solid particles or liquid droplets suspended in the air, has been shown to be associated with a number of health-related outcomes such as cancer, cardiovascular disease (CVD), and mortality [1]. For example, the International Agency for Research on Cancer has classified PM as a Group 1 carcinogen [2,3]. This is in part due to a large number of previous studies that demonstrated that increasing exposure to both long-term and short-term PM was associated with increased risk for cancer development [4]. Similarly, there is substantial evidence depicting higher risk for CVD upon elevated exposure to long-term and short-term PM levels among the general population [5]. While it is generally well-accepted that PM has a number of detrimental effects on health, recent focus has been on identifying susceptible populations to PM-related outcomes [6]. One such possible susceptible population are cancer survivors.

With recent advances in cancer management, the prevalence of cancer survivors, those who successfully received treatment for and survived cancer, is increasing globally. However, cancer survivors are at higher risk for developing subsequent serious illnesses, such as CVD. Recently, it has been shown that cancer survivors are at higher risk for developing CVD compared to those without cancer [7]. As cancer survivors are at increased risk for CVD events, it is of clinical importance to identify factors associated with CVD development among cancer survivors. Therefore, the fact that cancer survivors are at higher risk for CVD, as well as PM being a risk factor for both cancer and CVD development, indicates the possibility of PM being a risk factor for CVD among cancer survivors. Recently, it has been demonstrated that chronic, long-term exposure to higher levels of PM was associated with increased CVD risk among cancer survivors [8]. Moreover, a previous study has demonstrated that cancer survivors are more susceptible to long-term PM driven cardiopulmonary mortality compared to the general population [9]. In contrast to long-term PM exposure, there is a relative lack of evidence on whether short-term exposure to high levels of PM could lead to CVD events among cancer survivors.

Therefore, in this time-stratified case-crossover study using a nationwide administrative data, we aimed to determine the association of short-term PM exposure with CVD events.

## 2. Materials and Methods

The National Health Insurance Service (NHIS), which provides health insurance for all citizens in South Korea, covers nearly all forms of health services. Due to this, the NHIS has health administrative records of nearly the entire population of South Korea in the form of health claims data [10]. A part of this health claims data is provided for research purposes. The available data provided by the NHIS include information on sociodemographic factors, all inpatient and outpatient department visits, pharmaceutical drug prescriptions, and results from health screening examinations [10]. Multiple large-scale epidemiology studies have previously used the NHIS database, and its validity is described in detail elsewhere [11].

Daily PM levels categorized according to size (PM10 for PM < 10 μm, PM2.5 for PM < 2.5 μm, and PM2.5–10 for PM 2.5–10 μm in diameter) were derived from the Air Korea database, which was constructed from the National Ambient Air Monitoring System (NAMIS). The NAMIS collects daily air pollution levels and other weather-related information from more than 280 atmospheric monitoring sites located nationwide, with at least one monitoring site located in approximately 280 administrative districts in South Korea. The area of the administrative districts, which are a unit of municipality in South Korea, ranged between 2.8 and 775.0 km^2^, with the mean (standard deviation, SD) area being 55.1 (79.9) km^2^ [12]. Based on the administrative district, daily mean PM10, PM2.5, and PM2.5–10 levels were determined, after which the air pollution information was linked on an individual level based on each participant’s area of residence. Therefore, daily mean levels of PM10, PM2.5, and PM2.5–10 for each participant were determined according to their area of residence. A number of previous studies have used the NAMIS database in conjuncture with the NHIS data [8,13].

We extracted newly diagnosed cancer patients during the period 2006–2013. Cancer patients who survived for at least 5 years were defined as 5-year cancer survivors during the period 2011–2018. Among them, 23,044 cancer survivors with subsequent CVD events after the 5-year mark of cancer survival were determined. Those residing in areas without PM values (n = 180) were excluded, resulting in 22,864 5-year cancer survivors with subsequent CVD events during the period 2015–2018. Based on the CVD event (case) dates, referent periods were defined as dates in the same day of the week within the same month (e.g., all Monday dates for the month of March in 2017). Therefore, three or four referent dates were matched for each case (CVD) date, resulting in a total of 78,712 referent periods for 22,864 case periods. Based on the combined 101,576 case and referent periods, a time-stratified case-crossover design was used to determine the association of daily PM levels with short-term CVD events. Detailed descriptions of the case-crossover design are described elsewhere [14,15]. The time-stratified case-crossover design for determining short-term health-effects of PM exposure was derived from a previous study [16].

PM10, PM2.5, and PM2.5–10 levels were determined based on the case and reference dates. The mean daily PM level on the date of CVD event or its matched referent dates were defined as lag0. PM levels 1–3 days prior to the case or reference dates were defined as lag1, lag2, and lag3, respectively. The mean PM level over the 4-day period between the date of and 3 days prior to the case or referent date was defined as lag0–3. After determining lag0, lag1, lag2, lag3, and lag0-3 values, the PM levels were grouped according to quartiles of increasing PM levels, with the 1st (lowest, 0–25 percentile), 2nd (26–50 percentile), 3rd (51–75 percentile), and 4th (highest, 76–100 percentile) quartiles having increasing levels of PM for each lag period.

The operational definition for CVD, which was defined as being hospitalized for coronary heart disease (CHD) or stroke for 2 or more days, was adapted from a previous study that also used the NHIS database for identifying CVD events [11]. The diagnosis codes for CHD and stroke were based on the International Classification of Diseases, Tenth Edition (ICD-10). The ICD-10 codes for CHD (I20–I25) and stroke (I60–I69) were in line with those used by the American Heart Association [17]. Cancer patients were defined as those diagnosed with cancer (ICD-10 codes: C00–C97) with the critical condition code for cancer [8,18] The critical condition code for cancer is applied upon pathological confirmation of cancer, after which the patient benefits from significant reductions in cancer-related treatment costs.

Conditional logistic regression analysis was conducted to determine the adjusted odds ratios (aORs) and 95% confidence intervals (CIs) for short-term CVD events according to PM10, PM2.5, and PM2.5–10 levels among 5-year cancer survivors. Interactions of PM with other measures of PM and mean daily temperature were assessed in the association of PM with CVD incidence. No significant interactions were observed between PM2.5 with PM10, PM2.5 with PM2.5–10, PM10 with PM2.5–10, as well as between PM with mean daily temperature (all *p* for interaction > 0.05). Based upon this, a one-pollutant model was used to determine the association of short-term PM with CVD incidence. Upon regression analysis, mean daily temperature (categorical, 1st, 2nd, 3rd, and 4th quartiles), determined from the NAMIS Air Korea database, was adjusted for. Mean daily temperature was adjusted for via quartiles of the mean daily temperature value on the lag day. CVD odds were determined with the 1st (lowest) quartile group being the reference. *p* for trend was determined by assessing CVD odds per 1 quartile (thus, 25 percentile) increase in PM. Stratified analysis on the association of PM with acute CVD events among 5-year cancer survivors according to subgroups of age, sex, and household income was conducted. Household income was derived from the health insurance premium. Finally, the association of PM with short-term CHD and stroke events among 5-year cancer survivors was determined.

Statistical significance was defined upon a *p* value of <0.05 in a two-sided manner. All data collection and statistical analyses were conducted using SAS Enterprise Guide 7.1 (SAS Institute, Cary, NC, USA). Upon statistical analysis, the “proc logistic” command with the “stratified” option was used to fit the conditional logistic regression model.

## 3. Results

Table 1 depicts the descriptive characteristics of the study population. The mean (SD) PM10, PM2.5, and PM2.5–10 values for 22,864 5-year cancer survivors with CVD events were 44.9 (25.3) μg/m^3^, 25.1 (14.2) μg/m^3^, and 19.8 (17.1) μg/m^3^, respectively. The mean (SD) daily temperature was 12.9 (10.3) °C. The mean (SD) age for the study population was 71.7 (10.8) years. The number of participants (%) who were men or women were 12,750 (55.8) or 10,114 (44.2), respectively. Finally, 41.8% of the participants were within the highest quartile of household income.

**Figure 1:**
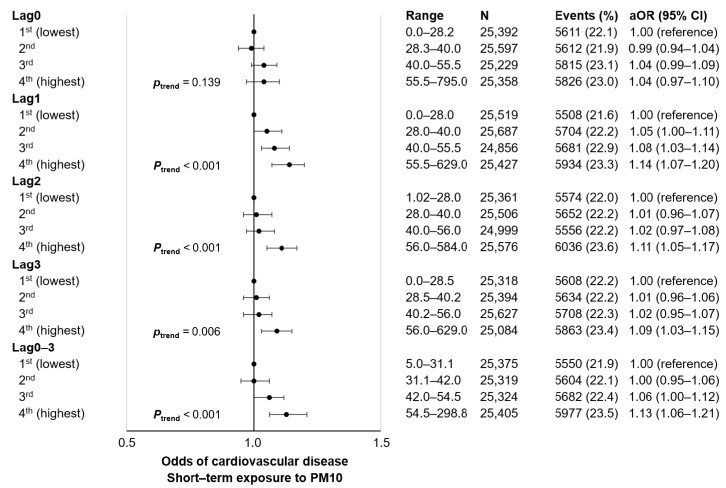
Association of short-term exposure to PM10 with cardiovascular disease events among 5-year cancer survivors. Adjusted odds ratio calculated by conditional logistic regression after adjustment for mean daily temperature (categorical, 1st, 2nd, 3rd, and 4th quartiles). Acronyms: N, number of participants; aOR, adjusted odds ratio; CI, confidence interval; PM, particulate matter.

The association of short-term PM10 exposure among 5-year cancer survivors with subsequent CVD events is shown in Figure 1 and Table S1. Compared to those within the 1st (lowest) quartile of lag1 PM10, those within the 3rd (aOR 1.08, 95% CI 1.03–1.14) and 4th (aOR 1.14, 95% CI 1.07–1.20) quartiles of lag1 PM10 had higher CVD events. Similarly, the 4th quartiles of lag2 (aOR 1.11, 95% CI 1.05–1.17) and lag3 (aOR 1.09, 95% CI 1.03–1.15) PM10 were associated with higher short-term CVD incidence compared to the respective lowest quartiles of PM10 exposure. Compared to the 1st quartile of lag0-3 PM10, the 4th quartile (aOR 1.13, 95% CI 1.06–1.21) was associated with higher CVD events. Finally, increasing quartiles of lag1, lag 2, lag3, and lag0–3 PM10 were associated with greater CVD odds in a dose-responsive manner (all *p* for trend < 0.05).

**Figure 2:**
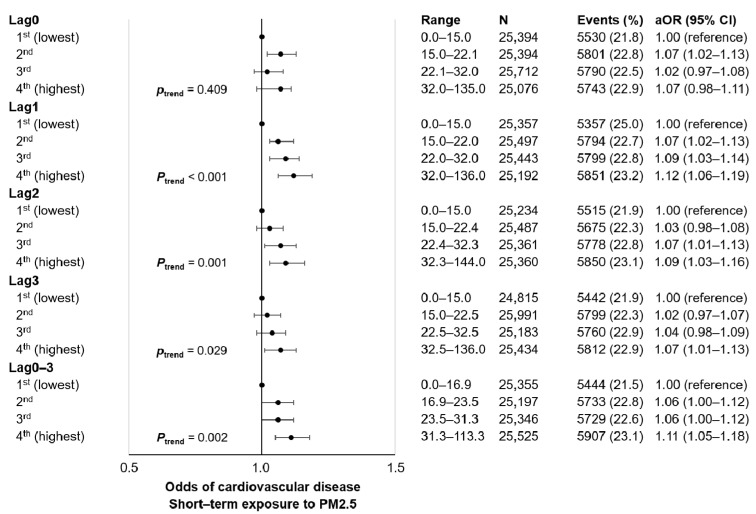
Association of short-term exposure to PM2.5 with cardiovascular disease events among 5-year cancer survivors. Adjusted odds ratio calculated by conditional logistic regression after adjustment for mean daily temperature (categorical, 1st, 2nd, 3rd, and 4th quartiles). Acronyms: N, number of participants; aOR, adjusted odds ratio; CI, confidence interval; PM, particulate matter.

The short-term CVD odds according to PM2.5 are depicted in Figure 2 and Table S2. Compared to the lowest quartile of lag1 PM2.5 exposure, the 2nd (aOR 1.07, 95% CI 1.02–1.13), 3rd (aOR 1.09, 95% CI 1.03–1.14), and 4th (aOR 1.12, 95% CI 1.06–1.19) quartiles of lag1 PM2.5 were associated with higher odds for short-term CVD events. Similarly, 3rd (aOR 1.07, 95% CI 1.01–1.13) and 4th (aOR 1.09, 95% CI 1.03–1.16) quartiles of lag2 PM2.5 were associated with higher CVD event rates compared to the lowest quartile of lag2 PM2.5. The highest quartile of lag0-3 PM2.5 (aOR 1.11, 95% CI 1.05–1.18) had higher odds for CVD compared to the 1st quartile. Finally, increasing levels of lag1, lag2, lag3, and lag0–3 PM2.5 were associated with higher CVD events in a dose-responsive manner (all *p* for trend <0.05). There was a tendency towards higher CHD and stroke odds upon increasing levels of short-term PM2.5 exposure, as shown in Supplementary Table S3.

**Figure 3:**
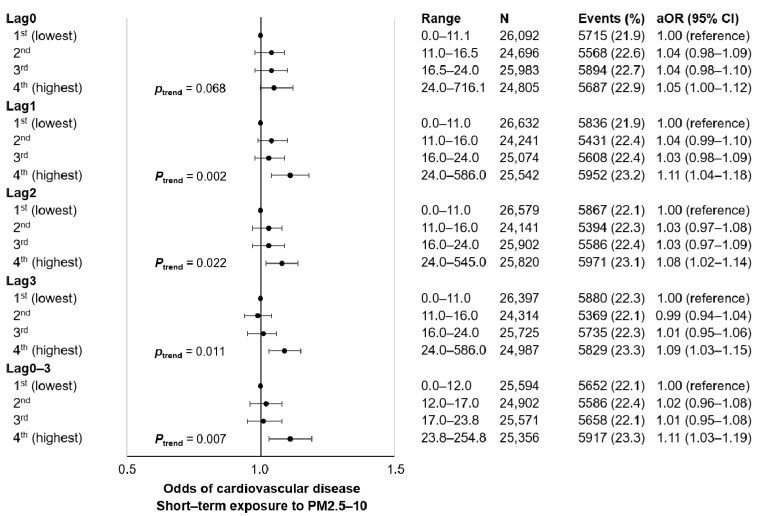
Association of short-term exposure to PM2.5-10 with cardiovascular disease events among 5-year cancer survivors. Adjusted odds ratio calculated by conditional logistic regression after adjustment for mean daily temperature (categorical, 1st, 2nd, 3rd, and 4th quartiles). Acronyms: N, number of participants; aOR, adjusted odds ratio; CI, confidence interval; PM, particulate matter.

Figure 3 and Table S3 depicts the association of short-term PM2.5–10 exposure with CVD among cancer survivors. The 4th quartiles of lag0 (aOR 1.05, 95% CI 1.00–1.12), lag1 (aOR 1.11, 95% CI 1.04–1.18), lag2 (aOR 1.08, 95% CI 1.02–1.14), lag3 (aOR 1.09, 95% CI 1.03–1.15), and lag0–3 (aOR 1.11, 95% CI 1.03–1.19) PM2.5–10 were associated with higher CVD incidence compared to the respective 1st quartiles. Increasing quartiles of lag1, lag2, lag3, and lag0–3 PM2.5–10 were associated with higher CVD events (all *p* for trend <0.05).

Table 2 shows the results from the stratified analysis on the association of short-term PM exposure with CVD among 5-year cancer survivors according to subgroups of age, sex, and household income. The 4th quartiles of lag0–3 PM10 for both men (aOR 1.14, 95% CI 1.04–1.24) and women (aOR 1.13, 95% CI 1.03–1.25) had higher CVD events compared to the 1st quartile. Similarly, the 4th quartiles of both the upper (aOR 1.14, 95% CI 1.05–1.24) and lower (aOR 1.12, 95% CI 1.01–1.25) halves of household income were associated with higher CVD compared to the respective 1st quartiles. The higher odds of CVD upon greater exposure to lag0–3 PM2.5 was preserved according to subgroups of age, sex, and household income. Table S4 depicts the association of PM with CHD or stroke among cancer survivors. Higher levels of PM10, PM2.5, and PM2.5-10 were associated with higher odds for CHD and stroke.

## 4. Discussion

In summary, short-term exposure to higher levels of PM10, PM2.5, and PM2.5–10 was associated with increased CVD events among 5-year cancer survivors. This association of higher CVD risk with increasing levels of short-term PM did not appear to be modified significantly according to age, sex, and household income. To our knowledge, this was the first study to demonstrate that higher short-term PM exposure was associated with higher CVD risk among 5-year cancer survivors.

Although we could not find any previous studies on the association of short-term PM exposure with CVD among cancer survivors, long-term and short-term PM exposure has previously been associated with the development of both CVD and cancer risk among the general population. Recently, a cohort study using the US National Health Interview Survey data showed that higher levels of PM2.5 were associated with increased risk for heart disease mortality [19]. Similarly, higher levels of short-term PM10 and PM2.5 exposure were shown to be associated with higher risk of stroke using the health professional’s follow-up study [16]. Recent meta-analysis studies have shown that long-term exposure to higher levels of was associated with significantly higher risk for lung cancer [4,20]. A previous study demonstrated that long-term exposure to traffic-related air pollution among patients with a history of myocardial infarction was associated with cancer risk [21]. Finally, it has been shown that long-term exposure to PM2.5 was associated with increased CVD risk among cancer survivors [8]. The results from our study add to these previous studies by showing that short-term exposure to higher levels of PM10, PM2.5, and PM2.5–10 was associated with higher CVD events among 5-year cancer survivors.

While the harmful effects of PM on cardiovascular health among the general population is generally well-accepted, there has been an emphasis on identifying susceptible populations on the detrimental effects of PM [6]. Determining such susceptible populations may lead to public health and preventive measures targeting certain populations who are especially susceptible to air pollution, particularly acute, short-term increases in PM exposure. Previously, it has been demonstrated that children, older adults, and those with low income are at higher risk for PM-related health effects [22,23,24]. Moreover, obese subjects, as well as those with pre-existing diseases such as CVD, respiratory disease, and diabetes have shown to be susceptible to the detrimental effects of PM [25,26,27]. However, there has been a lack of evidence on whether cancer survivors were also susceptible to PM-related health effects. This is of particular importance as cancer survivors have previously been shown to be at greater risk for a number of health-related outcomes including secondary primary cancer and CVD [7,28]. Moreover, it has been recently demonstrated that long-term exposure to PM2.5 was associated with higher cardiopulmonary mortality among cancer survivors (relative risk [RR] 1.25, 95% CI 1.21–1.30, per 10 μg/m^3^ increase) [9], which was higher than that from the general population (RR 1.11, 95% CI 1.08–1.14, per 10 μg/m^3^ increase) [29]. This previous study appears to suggest that cancer survivors are more susceptible to PM driven long-term cardiopulmonary risk compared to the general population. Therefore, it may be reasonable to expect a similar association for short-term PM exposure with CVD among cancer survivors compared to the general population. Thus, the increased CVD risk upon exposure to high levels of short-term PM among cancer survivors shown in our results could contribute to the implementation of certain public health or clinical preventive measures for cancer survivors.

Multiple possible mechanisms may contribute to the development of CVD upon exposure to high levels of PM [30]. First, PM inhalation may lead to lung inflammation, in which alveolar macrophages and inflammatory cytokines reacting to PM particles induce systemic inflammation [31,32]. Second, ingestion of PM particles via the gastrointestinal tract may result in gut inflammation and gut microbiome, which in turn could result in alveolocapillary translocation, leading to inflammation [33,34]. Third, PM particles may activate the hypothalamic-pituitary-adrenal axis, increasing the sympathetic nervous system and elevating catecholamine levels [35,36]. Systemic inflammation and elevated catecholamines may then lead to increased blood pressure, irregular heart rhythm, endothelial dysfunction, and acute phase response [37,38]. Ultimately, repeated stimuli of these subclinical changes by PM particles may result in acute atherosclerotic events.

Multiple limitations must be considered upon the interpretation of our results. First, while short-term PM exposure was measured on an individual level, PM exposure was nonetheless based on the daily PM levels according to the area of residence for each subject. Therefore, the short-term PM exposure defined in this study may not completely reflect each participant’s actual PM exposure. While multiple large-scale epidemiological studies using the NHIS database have previously used area of residence PM exposure, there is a need for future studies with a more accurate measure of short-term PM exposure [8,13]. Second, the operational definitions for cancer and CVD were not validated via medical chart records. The operational definitions for cancer and CVD used in this study has previously been used in other studies that also used the NHIS database [11,18]. Nonetheless, studies that use data with medical chart record-validated outcomes for cancer and CVD are needed. Third, we were unable to directly determine whether the association of short-term PM exposure with CVD among cancer survivors was particularly higher compared to that of the general population. While a previous study has suggested cancer survivors are at greater risk for cardiopulmonary mortality upon long-term PM exposure compared to the general population [9], whether cancer survivors are more susceptible to short-term PM driven CVD events should be investigated in future studies. Fourth, we were unable to adjust for time-varying variables other than temperature such as humidity due to the lack of information. While humidity may be associated with air pollutant concentration [39], little evidence suggests humidity alters short-term CVD risk [16]. Nonetheless, future studies that take into consideration other time-varying factors such as humidity are needed. Finally, we could not conduct detailed analysis based on cancer types due to the lack of enough subjects. While previous studies have shown oropharyngeal, rectal, and breast cancer survivors were associated with higher mortality risk upon long-term PM exposure [9,40,41], whether these cancer types are also susceptible to PM driven short-term CVD incidence is yet unknown. Future studies that determine the health-related short-term effects of PM exposure according to cancer types among cancer survivors would be beneficial.

Despite these limitations, this study has a number of strengths. First, there is a relatively low possibility of bias from individual factors, such as age and sex, due to the time-stratified case-crossover design in which comparisons are made within each subject among different (case and referent periods) time frames. This is of particular importance in cancer survivorship studies, as the database lacked detailed clinical information such as cancer management regimens and the pathological stage. While such factors could lead to bias when comparing between subjects, such as in cohort studies, case-crossover studies are relatively less susceptible to confounding effects of such characteristics [15]. Nonetheless, we conducted stratified analysis on the association of short-term PM with CVD risk among cancer survivors according to age, sex, and household income. The results from the stratified analysis appear to suggest age, sex, and household income did not appear to significantly modify the association of short-term PM exposure with CVD events among cancer survivors. Second, we used a nationwide database which includes nearly all cancer survivors in South Korea, thus enhancing the generalizability of our results. Finally, we measured short-term PM2.5–10 concentrations as well as PM2.5 and PM10, demonstrating coarse PMs may also elevate short-term CVD risk among cancer survivors.

## 5. Conclusions

Being exposed to high levels of short-term PM10 and PM2.5 may lead to acute CVD events among cancer survivors. Therefore, cancer survivors may be susceptible to the acute CVD-risk increasing effects of daily PM. Cancer survivors who reduce short-term exposure to high levels of PM10 and PM2.5 may benefit from reduced risk of acute cardiovascular events. Future studies must investigate whether intervention methods aimed at reducing short-term PM exposure actually lead to lower CVD events in cancer survivors.

## Figures and Tables

**Table 1:** Descriptive characteristics of the study population.

	Cardiovascular Disease	Coronary Heart Disease	Stroke
Number of participants	22,864	8306	14,558
Lag0–3 PM10, μg/m^3^			
Mean (SD)	44.9 (25.3)	44.6 (24.9)	45.1 (25.4)
Range (minimum-maximum)			
1st quartile	5.0–31.1	5.0–31.1	5.0–31.1
2nd quartile	31.1–42.0	31.1–42.0	31.1–42.0
3rd quartile	42.0–54.5	42.0–54.5	42.0–54.5
4th quartile	54.5–298.8	54.5–289.9	54.5–298.8
Lag0–3 PM2.5, μg/m^3^			
Mean (SD)	25.1 (14.2)	25.0 (14.4)	25.2 (14.0)
Range (minimum-maximum)			
1st quartile	0.0–16.9	0.0–16.9	0.0–16.9
2nd quartile	16.9–23.5	16.9–23.5	16.9–23.5
3rd quartile	23.5–31.3	23.5–31.3	23.5–31.3
4th quartile	31.3–113.3	31.3–113.3	31.3–109.5
Lag0–3 PM2.5–10, μg/m^3^			
Mean (SD)	19.8 (17.1)	19.6 (16.4)	20.0 (17.6)
Range (minimum-maximum)			
1st quartile	0.0–12.0	0.0–12.0	0.0–12.0
2nd quartile	12.0–17.0	12.0–17.0	12.0–17.0
3rd quartile	17.0–23.8	17.0–23.8	17.0–23.8
4th quartile	23.8–254.8	23.8–245.9	23.8–254.8
Temperature, °C, mean (SD)	12.9 (10.3)	12.8 (10.3)	12.9 (10.3)
Age, years, mean (SD)	71.7 (10.8)	70.5 (10.3)	72.5 (11.1)
Sex, N (%)			
Men	12,750 (55.8)	5178 (62.3)	7572 (52.0)
Women	10,114 (44.2)	3128 (37.7)	6986 (48.0)
Household income, quartiles, N (%)			
1st (highest)	9564 (41.8)	3437 (41.4)	6127 (42.1)
2nd	4033 (17.6)	1519 (18.3)	2514 (17.3)
3rd	2796 (12.2)	1015 (12.2)	1781 (12.2)
4th (lowest)	6471 (28.3)	2335 (28.1)	4136 (28.4)

Acronyms: PM, particulate matter; SD, standard deviation; N, number of participants.

**Table 2:** Stratified analysis on the association of short-term PM with cardiovascular disease among 5-year cancer survivors according to subgroups of age, sex, and household income.

	Adjusted Odds Ratio, 95% Confidence Interval				
	PM, Quartiles				
	1st (Lowest)	2nd	3rd	4th (Highest)	*p* for Trend
PM10, lag 0–3					
Age, years					
<65	1.00 (reference)	1.01 (0.91–1.13)	1.04 (0.92–1.17)	1.09 (0.96–1.24)	0.173
≥65	1.00 (reference)	1.00 (0.94–1.06)	1.06 (0.99–1.13)	1.15 (1.06–1.24)	<0.001
Sex					
Men	1.00 (reference)	1.03 (0.95–1.10)	1.07 (0.99–1.16)	1.14 (1.04–1.24)	0.003
Women	1.00 (reference)	0.97 (0.89–1.05)	1.05 (0.95–1.14)	1.13 (1.03–1.25)	0.004
Household income					
Upper half	1.00 (reference)	1.01 (0.94–1.08)	1.08 (1.00–1.16)	1.14 (1.05–1.24)	<0.001
Lower half	1.00 (reference)	0.99 (0.91–1.08)	1.02 (0.93–1.12)	1.12 (1.01–1.25)	0.015
PM2.5, lag 0–3					
Age, years					
<65	1.00 (reference)	1.10 (0.98–1.21)	1.17 (1.04–1.30)	1.15 (1.01–1.29)	0.025
≥65	1.00 (reference)	1.05 (0.99–1.12)	1.02 (0.96–1.09)	1.11 (1.04–1.19)	0.014
Sex					
Men	1.00 (reference)	1.07 (1.00–1.15)	1.03 (0.95–1.11)	1.09 (1.00–1.18)	0.122
Women	1.00 (reference)	1.06 (0.98–1.15)	1.09 (1.00–1.20)	1.16 (1.06–1.27)	0.002
Household income					
Upper half	1.00 (reference)	1.06 (0.99–1.13)	1.04 (0.97–1.12)	1.12 (1.04–1.22)	0.010
Lower half	1.00 (reference)	1.08 (0.99–1.16)	1.08 (0.99–1.18)	1.11 (1.01–1.23)	0.044
PM2.5–10, lag 0–3					
Age, years					
<65	1.00 (reference)	1.04 (0.93–1.16)	1.02 (0.90–1.16)	1.13 (0.98–1.28)	0.119
≥65	1.00 (reference)	1.01 (0.95–1.08)	1.01 (0.94–1.08)	1.10 (1.02–1.19)	0.026
Sex					
Men	1.00 (reference)	1.04 (0.97–1.12)	1.10 (1.01–1.19)	1.13 (1.02–1.26)	0.005
Women	1.00 (reference)	0.98 (0.90–1.07)	0.91 (0.82–1.00)	1.07 (0.96–1.19)	0.386
Household income					
Upper half	1.00 (reference)	1.02 (0.95–1.10)	1.01 (0.93–1.10)	1.11 (1.02–1.20)	0.042
Lower half	1.00 (reference)	1.01 (0.93–1.10)	1.01 (0.92–1.12)	1.12 (1.00–1.24)	0.065

Adjusted odds ratios calculated by conditional logistic regression after adjustment for mean daily temperature (categorical, 1st, 2nd, 3rd, and 4th quartiles). Acronyms: PM, particulate matter.

## Data Availability

The authors do not have the authority to share the data. The NHIS has a strict confidentiality policy in which researchers are not allowed to share the data as a part of privacy protection procedures. Access to the data can be applied at the NHIS website.

## Supplementary Materials

The following are available online at https://www.mdpi.com/article/10.3390/ijerph18157996/s1, Table S1: Association of short-term exposure to PM10 with cardiovascular disease events among 5-year cancer survivors, Table S2: Association of short-term exposure to PM2.5 with cardiovascular disease events among 5-year cancer survivors, Table S3: Association of short-term exposure to PM2.5-10 with cardiovascular disease events among 5-year cancer survivors, Table S4: Association of short-term PM with coronary heart disease or stroke among 5-year cancer survivors.
