# Supplementary material for: Association of Short-Term Particulate Matter Exposure among 5-Year Cancer Survivors with Incident Cardiovascular Disease: A Time-Stratified Case-Crossover Study
Source: Int J Environ Res Public Health. 2021 Jul 28;18(15):7996. doi: 10.3390/ijerph18157996 (PMC8345681; doi:10.3390/ijerph18157996)
Supplement: Supplementary file 1 [file ijerph-18-07996-s001.zip › ijerph-1283289-supplementary.pdf]

International Journal of *Environmental Research and Public Health*

Article

Association of Short-Term Particulate Matter Exposure among 5-Year Cancer Survivors with Incident Cardiovascular Disease: A Time-Stratified Case-Crossover Study

Seulggie Choi [1], Kyae Hyung Kim [2], Daein Choi [3], Seogsong Jeong [1], Kyuwoong Kim [4], Jooyoung Chang [1], Sung Min Kim [1], Seong Rae Kim [5], Yoosun Cho [6], Gyeongsil Lee [2], Joung Sik Son [7] and Sang Min Park [2,8,*]

1 Department of Biomedical Sciences, Graduate School, Seoul National University, Seoul 03080, Korea; seulggie@gmail.com (S.C.); seogsongjeong@gmail.com (S.J.); joomyjoo@gmail.com (J.C.); ksm9904@naver.com (S. M.K.)
2 Department of Family Medicine, Seoul National University Hospital, Seoul 03080, Korea; truwhat@gmail.com (K.H.K.); gespino1_gs@gmail.com (G.L.)
3 Department of Internal Medicine, Mount Sinai Beth Israel, Icahn School of Medicine at Mount Sinai, New York, NY 10029, USA; daeinchoi.md@gmail.com
4 National Cancer Control Institute, National Cancer Center, Goyang 10408, Korea; kwkim238@gmail.com
5 College of Medicine, Seoul National University Hospital, Seoul 03080, Korea; sungkim20@snu.ac.kr
6 Total Healthcare Center, Kangbuk Samsung Hospital, School of Medicine, Sungkyunkwan University, Seoul 06351, Korea; misslonghorn46@gmail.com
7 Department of Family Medicine, Korea University Guro Hospital, Seoul 08308, Korea; medical114@naver.com
8 Department of Biomedical Sciences, College of Medicine, Seoul National University, Seoul 03080, Korea
* Correspondence: smpark.snuh@gmail.com; Tel.: +82-2-2072-3331

Citation: Choi, S.; Kim, K.H.; Choi, D.; Jeong, S.; Kim, K.; Chang, J.; Kim, S.M.; Kim, S.R.; Cho, Y.; Lee, G.; et al. Association of Short-Term Particulate Matter Exposure among 5-Year Cancer Survivors with Incident Cardiovascular Disease: A Time-Stratified Case-Crossover Study. *Int. J. Environ. Res. Public Health* **2021**, *18*, 7996. https://doi.org/10.3390/ijerph18157996

Academic Editor: Paul B. Tchounwou

Received: 17 June 2021
Accepted: 27 July 2021
Published: 28 July 2021

Publisher's Note: MDPI stays neutral with regard to jurisdictional claims in published maps and institutional affiliations.

Copyright: © 2021 by the authors. Licensee MDPI, Basel, Switzerland. This article is an open access article distributed under the terms and conditions of the Creative Commons Attribution (CC BY) license (http://creativecommons.org/licenses/by/4.0/).

Table S1. Association of short-term exposure to PM10 with cardiovascular disease events among 5-year cancer survivors.

	PM10, quartiles				
	**1st (lowest)**	**2nd**	**3rd**	**4th (highest)**	***p* for trend**
Lag 0					
Range, μg/m³	0.0-28.2	28.3-40.0	40.0-55.5	55.5-795.0	
Number of participants	25,392	25,597	25,229	25,358	
Events (%)	5,611 (22.1)	5,612 (21.9)	5,815 (23.1)	5,826 (23.0)	
aOR (95% CI)	1.00 (reference)	0.99 (0.94-1.04)	1.04 (0.99-1.09)	1.04 (0.97-1.10)	0.139
Lag 1					
Range, μg/m³	0.0-28.0	28.0-40.0	40.0-55.5	55.5-629.0	
Number of participants	25,519	25,687	24,856	25,427	
Events (%)	5,508 (21.6)	5,704 (22.2)	5,681 (22.9)	5,934 (23.3)	
aOR (95% CI)	1.00 (reference)	1.05 (1.00-1.11)	1.08 (1.03-1.14)	1.14 (1.07-1.20)	<0.001
Lag 2					
Range, μg/m³	1.0-28.0	28.0-40.0	40.0-56.0	56.0-584.0	
Number of participants	25,361	25,506	24,999	25,576	
Events (%)	5,574 (22.0)	5,652 (22.2)	5,556 (22.2)	6,036 (23.6)	
aOR (95% CI)	1.00 (reference)	1.01 (0.96-1.07)	1.02 (0.97-1.08)	1.11 (1.05-1.17)	<0.001
Lag 3					
Range, μg/m³	0.0-28.5	28.5-40.2	40.2-56.0	56.0-629.0	
Number of participants	25,318	25,394	25,627	25,084	
Events (%)	5,608 (22.2)	5,634 (22.2)	5,708 (22.3)	5,863 (23.4)	
aOR (95% CI)	1.00 (reference)	1.01 (0.96-1.06)	1.02 (0.95-1.07)	1.09 (1.03-1.15)	0.006
Lag 0-3 (average)					
Range, μg/m³	5.0-31.1	31.1-42.0	42.0-54.5	54.5-298.8	
Number of participants	25,375	25,319	25,324	25,405	
Events (%)	5,550 (21.9)	5,604 (22.1)	5,682 (22.4)	5,977 (23.5)	
aOR (95% CI)	1.00 (reference)	1.00 (0.95-1.06)	1.06 (1.00-1.12)	1.13 (1.06-1.21)	<0.001

Adjusted odds ratios calculated by conditional logistic regression after adjustment for mean daily temperature (categorical, 1st, 2nd, 3rd, and 4th quartiles). Acronyms: PM10, particulate matter 10; aOR, adjusted odds ratio; CI, confidence interval.

Table S2. Association of short-term exposure to PM2.5 with cardiovascular disease events among 5-year cancer survivors.

	**PM2.5, quartiles**				
	**1st (lowest)**	**2nd**	**3rd**	**4th (highest)**	***p* for trend**
Lag 0					
Range, μg/m³	0.0-15.0	15.0-22.1	22.1-32.0	32.0-135.0	
Number of participants	25,394	25,394	25,712	25,076	
Events (%)	5,530 (21.8)	5,801 (22.8)	5,790 (22.5)	5,743 (22.9)	
aOR (95% CI)	1.00 (reference)	1.07 (1.02-1.13)	1.02 (0.97-1.08)	1.07 (0.98-1.11)	0.409
Lag 1					
Range, μg/m³	0.0-15.0	15.0-22.0	22.0-32.0	32.0-136.0	
Number of participants	25,357	25,497	25,443	25,192	
Events (%)	5,357 (25.0)	5,794 (22.7)	5,799 (22.8)	5,851 (23.2)	
aOR (95% CI)	1.00 (reference)	1.06 (1.03-1.12)	1.09 (1.03-1.14)	1.12 (1.06-1.19)	<0.001
Lag 2					
Range, μg/m³	0.0-15.0	15.0-22.4	22.4-32.3	32.3-144.0	
Number of participants	25,234	25,487	25,361	25,360	
Events (%)	5,515 (21.9)	5,675 (22.3)	5,778 (22.8)	5,850 (23.1)	
aOR (95% CI)	1.00 (reference)	1.03 (0.98-1.08)	1.07 (1.01-1.13)	1.09 (1.03-1.16)	0.001
Lag 3					
Range, μg/m³	0.0-15.0	15.0-22.5	22.5-32.5	32.5-136.0	
Number of participants	24,815	25,991	25,183	25,434	
Events (%)	5,442 (21.9)	5,799 (22.3)	5,760 (22.9)	5,812 (22.9)	
aOR (95% CI)	1.00 (reference)	1.02 (0.97-1.07)	1.04 (0.98-1.09)	1.07 (1.01-1.13)	0.029
Lag 0-3 (average)					
Range, μg/m³	0.0-16.9	16.9-23.5	23.5-31.3	31.3-113.3	
Number of participants	25,355	25,197	25,346	25,525	
Events (%)	5,444 (21.5)	5,733 (22.8)	5,729 (22.6)	5,907 (23.1)	
aOR (95% CI)	1.00 (reference)	1.06 (1.00-1.12)	1.06 (1.00-1.12)	1.11 (1.05-1.18)	0.002

Adjusted odds ratios calculated by conditional logistic regression after adjustment for mean daily temperature (categorical, 1st, 2nd, 3rd, and 4th quartiles). Acronyms: PM2.5, particulate matter 2.5; aOR, adjusted odds ratio; CI, confidence interval.

Table 3. Association of short-term exposure to PM2.5-10 with cardiovascular disease events among 5-year cancer survivors.

	PM2.5-10, quartiles				
	**1st (lowest)**	**2nd**	**3rd**	**4th (highest)**	***p* for trend**
Lag 0					
Range, μg/m³	0.0-11.1	11.0-16.5	16.5-24.0	24.0-716.1	
Number of participants	26,092	24,696	25,983	24,805	
Events (%)	5,715 (21.9)	5,568 (22.6)	5,894 (22.7)	5,687 (22.9)	
aOR (95% CI)	1.00 (reference)	1.04 (0.98-1.09)	1.04 (0.98-1.10)	1.05 (1.00-1.12)	0.068
Lag 1					
Range, μg/m³	0.0-11.0	11.0-16.0	16.0-24.0	24.0-586.0	
Number of participants	26,632	24,241	25,074	25,542	
Events (%)	5,836 (21.9)	5,431 (22.4)	5,608 (22.4)	5,952 (23.2)	
aOR (95% CI)	1.00 (reference)	1.04 (0.99-1.10)	1.03 (0.98-1.09)	1.11 (1.04-1.18)	0.002
Lag 2					
Range, μg/m³	0.0-11.0	11.0-16.0	16.0-24.0	24.0-545.0	
Number of participants	26,579	24,141	24,902	25,820	
Events (%)	5,867 (22.1)	5,394 (22.3)	5,586 (22.4)	5,971 (23.1)	
aOR (95% CI)	1.00 (reference)	1.03 (0.97-1.08)	1.03 (0.97-1.09)	1.08 (1.02-1.14)	0.022
Lag 3					
Range, μg/m³	0.0-11.0	11.0-16.0	16.0-24.0	24.0-586.0	
Number of participants	26,397	24,314	25,725	24,987	
Events (%)	5,880 (22.3)	5,369 (22.1)	5,735 (22.3)	5,829 (23.3)	

aOR (95% CI)	1.00 (reference)	0.99 (0.94-1.04)	1.01 (0.95-1.06)	1.09 (1.03-1.15)	0.011
Lag 0-3 (average)					
Range, μg/m^3	0.0-12.0	12.0-17.0	17.0-23.8	23.8-254.8	
Number of participants	25,594	24,902	25,571	25,356	
Events (%)	5,652 (22.1)	5,586 (22.4)	5,658 (22.1)	5,917 (23.3)	
aOR (95% CI)	1.00 (reference)	1.02 (0.96-1.08)	1.01 (0.95-1.08)	1.11 (1.03-1.19)	0.007

Adjusted odds ratios calculated by conditional logistic regression after adjustment for mean daily temperature (categorical, 1st, 2nd, 3rd, and 4th quartiles). Acronyms: PM2.5, particulate matter between 2.5-10; aOR, adjusted odds ratio; CI, confidence interval.

Table S4. Association of short-term PM with coronary heart disease or stroke among 5-year cancer survivors.

	Adjusted odds ratio, 95% confidence interval				
	PM, quartiles				
	**1st (lowest)**	**2nd**	**3rd**	**4th (highest)**	***p* for trend**
Coronary heart disease					
PM10					
Lag 0	1.00 (reference)	0.97 (0.89-1.06)	1.00 (0.91-1.09)	1.01 (0.92-1.11)	0.711
Lag 1	1.00 (reference)	1.04 (0.95-1.13)	1.03 (0.94-1.12)	1.01 (0.92-1.11)	0.913
Lag 2	1.00 (reference)	1.01 (0.92-1.09)	0.98 (0.90-1.07)	0.97 (0.89-1.07)	0.489
Lag 3	1.00 (reference)	0.98 (0.90-1.07)	0.98 (0.90-1.07)	1.01 (0.92-1.11)	0.805
Lag 0-3	1.00 (reference)	0.98 (0.90-1.07)	0.96 (0.87-1.06)	1.01 (0.91-1.12)	0.891
PM2.5					
Lag 0	1.00 (reference)	1.04 (0.95-1.13)	0.98 (0.90-1.07)	1.06 (0.97-1.17)	0.435
Lag 1	1.00 (reference)	1.08 (0.99-1.17)	1.04 (0.95-1.14)	1.05 (0.96-1.15)	0.508
Lag 2	1.00 (reference)	1.05 (0.97-1.14)	1.03 (0.94-1.12)	1.03 (0.94-1.12)	0.748
Lag 3	1.00 (reference)	0.99 (0.91-1.08)	1.00 (0.92-1.09)	1.07 (0.98-1.17)	0.110
Lag 0-3	1.00 (reference)	1.09 (1.00-1.19)	1.07 (0.98-1.17)	1.08 (0.98-1.20)	0.228
PM2.5-10					
Lag 0	1.00 (reference)	1.05 (0.96-1.14)	0.98 (0.90-1.08)	1.00 (0.90-1.11)	0.694
Lag 1	1.00 (reference)	1.00 (0.92-1.09)	0.99 (0.91-1.09)	0.98 (0.89-1.08)	0.690
Lag 2	1.00 (reference)	1.06 (0.98-1.16)	1.00 (0.91-1.09)	0.94 (0.85-1.04)	0.131
Lag 3	1.00 (reference)	1.02 (0.94-1.11)	0.99 (0.91-1.09)	0.99 (0.89-1.09)	0.684
Lag 0-3	1.00 (reference)	0.97 (0.89-1.07)	0.91 (0.82-1.00)	0.91 (0.82-1.02)	0.073
Stroke					
PM10					
Lag 0	1.00 (reference)	1.00 (0.94-1.07)	1.07 (1.00-1.14)	1.04 (0.97-1.13)	0.111
Lag 1	1.00 (reference)	1.06 (0.99-1.13)	1.09 (1.02-1.17)	1.19 (1.10-1.28)	<0.001
Lag 2	1.00 (reference)	1.01 (0.95-1.08)	1.03 (0.96-1.11)	1.18 (1.10-1.27)	<0.001
Lag 3	1.00 (reference)	1.01 (0.95-1.08)	1.03 (0.96-1.10)	1.13 (1.05-1.22)	<0.001
Lag 0-3	1.00 (reference)	1.01 (0.95-1.08)	1.11 (1.03-1.20)	1.21 (1.12-1.31)	<0.001
PM2.5					

Lag 0	1.00 (reference)	1.09 (1.02-1.17)	1.04 (0.98-1.12)	1.04 (0.96-1.12)	0.667
Lag 1	1.00 (reference)	1.07 (1.00-1.14)	1.10 (1.03-1.18)	1.15 (1.07-1.23)	<0.001
Lag 2	1.00 (reference)	1.01 (0.95-1.07)	1.08 (1.01-1.16)	1.11 (1.03-1.19)	<0.001
Lag 3	1.00 (reference)	1.02 (0.96-1.09)	1.06 (0.99-1.13)	1.05 (0.98-1.13)	0.099
Lag 0-3	1.00 (reference)	1.05 (0.98-1.12)	1.04 (0.97-1.12)	1.14 (1.06-1.23)	0.002
PM2.5-10					
Lag 0	1.00 (reference)	1.03 (0.96-1.10)	1.08 (1.00-1.16)	1.10 (1.02-1.19)	0.008
Lag 1	1.00 (reference)	1.07 (1.00-1.15)	1.07 (0.99-1.15)	1.20 (1.12-1.30)	<0.001
Lag 2	1.00 (reference)	1.00 (0.94-1.07)	1.04 (0.97-1.12)	1.17 (1.08-1.26)	<0.001
Lag 3	1.00 (reference)	0.97 (0.91-1.04)	1.02 (0.95-1.09)	1.16 (1.07-1.25)	<0.001
Lag 0-3	1.00 (reference)	1.05 (0.97-1.12)	1.08 (1.00-1.17)	1.24 (1.14-1.36)	<0.001

Adjusted odds ratios calculated by conditional logistic regression after adjustment for mean daily temperature (categorical, 1st, 2nd, 3rd, and 4th quartiles). Acronyms: PM, particulate matter.
